# Fluorescence calibration standards made from broadband emitters encapsulated in polymer beads for fluorescence microscopy and flow cytometry

**DOI:** 10.1007/s00216-020-02664-y

**Published:** 2020-05-14

**Authors:** Katrin Hoffmann, Nithiya Nirmalananthan-Budau, Ute Resch-Genger

**Affiliations:** BAM Federal Institute for Materials Research and Testing, Richard-Willstaetter-Str. 11, 12489 Berlin, Germany

**Keywords:** Spectral calibration beads, Fluorescence microscopy, Dye, Polymer particle, Emission correction curve, Fluorescence standards

## Abstract

We present here the design and characterization of a set of spectral calibration beads. These calibration beads are intended for the determination and regular control of the spectral characteristics of fluorescence microscopes and other fluorescence measuring devices for the readout of bead-based assays. This set consists of micrometer-sized polymer beads loaded with dyes from the liquid Calibration Kit *Spectral Fluorescence Standards* developed and certified by BAM for the wavelength-dependent determination of the spectral responsivity of fluorescence measuring devices like spectrofluorometers. To cover the wavelength region from 400 to 800 nm, two new near-infrared emissive dyes were included, which were spectroscopically characterized in solution and encapsulated in the beads. The resulting set of beads presents the first step towards a new platform of spectral calibration beads for the determination of the spectral characteristics of fluorescence instruments like fluorescence microscopes, FCM setups, and microtiter plate readers, thereby meeting the increasing demand for reliable and comparable fluorescence data especially in strongly regulated areas, e.g., medical diagnostics. This will eventually provide the basis for standardized calibration procedures for imaging systems as an alternative to microchannel slides containing dye solutions previously reported by us.

**Electronic supplementary material:**

The online version of this article (10.1007/s00216-020-02664-y) contains supplementary material, which is available to authorized users.

**Figure:**
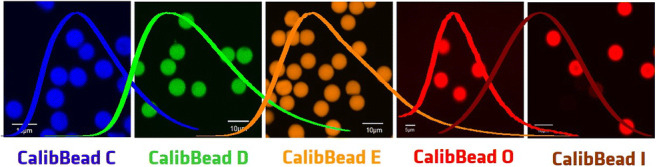
Graphical abstract

## Introduction

All fluorescence-based techniques generate signals that contain not only sample-related but also instrument-specific contributions. This limits the straightforward comparison of fluorescence data obtained, e.g., on the same instrument, but at different times, and particularly on different devices [1–7], and hampers quantification. Moreover, the recent trend to collect and provide reference data for all analytical techniques triggers the need for data bases that contain reliably corrected instrument-independent emission spectra of fluorescent reporters, probes, and sensors frequently used by the fluorescence community. In addition, fluorescence-based techniques like flow cytometry (FCM) and fluorescence microscopy applied in the life sciences and frequently also in regulated areas like medical diagnostics or pharmaceutical research require regular instrument calibration and performance validation for comparable and quantitative measurements.

To rule out instrumentation as a major source of variability of emission data, easy to use, accessible, and generally accepted fluorescence standards are needed together with validated procedures for the control of instrument specifications and long-term performance [1, 8, 9]. Such fluorescence standards, which can be physical devices like calibration lamps or chromophore-based chemical reference materials, should enable the determination of the relevant instrument parameters and instrument characteristics under application-relevant conditions as well as regular validation of instrument performance (IPV) to efficiently determine mandatory calibration intervals and to provide a regular basis for data quality [10]. For the determination of the spectral characteristics of fluorescence measuring instruments, which are the cause of instrument-specific data and make spectrally uncorrected fluorescence data not comparable across different instruments and laboratories, either calibration lamps [4] with a known wavelength dependence of their spectral radiance are employed, or chemical standards [11–13]. The latter are more user-friendly, closer to typically measured samples, and can be used under routine measurement conditions. Examples of chemical standards include solutions of molecular fluorophores with broad unstructured emission spectra such as the fluorophores F001–F005 present in the BAM Calibration Kit *Spectral Fluorescence Standards* or the luminescent glasses available from the National Institute for Standards and Technology (NIST) [11, 12, 14, 15]. The commercialized BAM Kit contains not only these five dyes with overlapping emission spectra [12] but also the custom-made software *LinkCorr* (updated meanwhile to *LinkKorrWin*) which calculates the wavelength-dependent spectral responsivity *s*(*λ*_em_) of the instrument to be calibrated from the Kit dye spectra measured with this instrument and the certified dye spectra included in *LinkCorr*. Thereby, *s*(*λ*_em_) or the inverse spectral responsivity curve *s*(*λ*_em_)^−1^, the emission correction curve, is obtained in a standardized manner. BAM recently also certified a multi-functional multi-emitter glass intended for use as day-to-day intensity standard that contains a mixture of different narrow band emitting lanthanide ions and is available in a cuvette-shaped and slide-shaped geometry [8]. Regular measurements of the intensity pattern of this standard can provide information on changes in the spectral characteristics of fluorescence measuring devices.

For FCM, a broad variety of fluorophore-stained polymer beads differing in emission color and intensity is available for the testing of the alignment, sensitivity, and other parameters of FCM instruments. These calibration tools are intended to facilitate the assessment of instrument performance to ensure reliable measurements and to improve the comparability of FCM experiments [16–20, 22] (https://www.thermofisher.com/de/de/home/references/newsletters-and-journals/bioprobes-journal-of-cell-biology-applications/bioprobes-70/fluorescent-microspheres-for-calibration.html), (https://www.sigmaaldrich.com/content/dam/sigma-aldrich/docs/Sigma/Datasheet/10/75194dat.pdf). Also for fluorescence microscopy, in addition to a very small number of relatively expensive structured calibration tools like the slide from Argolight [21, 22], different calibration beads have been commercialized to determine parameters like resolution *x*/*y*/*z*, intensity calibration, color adjustment, instrument alignment, and stability [20, 23, 24] (https://www.thermofisher.com/de/de/home/references/newsletters-and-journals/bioprobes-journal-of-cell-biology-applications/bioprobes-70/fluorescent-microspheres-for-calibration.html). However, these broadly used beads have been neither designed nor yet used to determine the spectral characteristics of fluorescence measuring devices, like spectral scanning fluorescence microscopes, spectral FCM setups, microtiter plate readers, and all types of integral measuring devices, although for fluorescence microscopic techniques and even FCM, new trends in instrument design focus increasingly on spectrally resolved measurements [25, 26]. To the best of our knowledge, the only exception presents micrometer-sized calibration beads containing luminescent lanthanide ion complexes, which were used for the spectral calibration of microscopes equipped with a spectrograph. The emission of these complexes between 550 and 750 nm, however, consists of a set of relative narrow bands as typical for lanthanides with their f-f transitions. This makes them unsuited for the determination of the wavelength-dependent spectral responsivity [20].

This motivated us to develop bead-based spectral standards with emission spectra covering the visible spectral region from about 400 to 800 nm that can be used as calibration set similarly like the dye solution-based Kit F001–F005. Such micrometer-sized calibration beads are much closer to eukaryotic and bacteria cells typically studied with FCM and fluorescence microscopy techniques as dye solutions. Moreover, fluorophore encapsulation in beads not only elegantly circumvents possible interactions between the dyes and the environment but also can enhance the fluorescence quantum yield, and thus brightness. This is, e.g., the case for fluorophores that contain flexible moieties, the rotation of which is linked to a non-radiative pathway of the excited singlet state as is the case, e.g., for many cyanine dyes [27, 28]. This, together with the generally improved stability of bead-encapsulated dyes, is particularly important for near-infrared (NIR) emissive dyes which tend to be less emissive and less stable as fluorophores with emission in the visible region [29]. Here, we present a proof-of-concept study to generate a set of candidate spectral calibration beads from different hydrophobic organic dyes, premanufactured micrometer-sized polystyrene (PS) particles, and previously developed swelling procedures [29–31].

## Materials and methods

### Materials

Polystyrene (PS) microparticles were purchased from Kisker Biotech GmbH & Co. KG. The dyes A to E (equalling F001 to F005 of the BAM Calibration Kit *Spectral Fluorescence Standards*) [4, 12] with emission in the visible region, the validation dye Y [12], and the NIR dyes O and I [29] were used without further purification. Tetrahydrofuran (THF) was of UV-spectroscopic grade and purchased from Sigma-Aldrich Co. (Germany).

### Bead staining/encapsulation of dyes

Carboxy-functionalized polystyrene (PS) particles with diameters of 8 μm were stained with the hydrophobic Kit dyes F001–F005 [4], resulting in the candidate calibration beads CBead A to CBead E. Following a protocol from Behnke et al. [29–32], 100 μL of the dye solution in THF with a dye concentration of 2 mM was added to 600 μL of an aqueous suspension of 3 mg of PS particles (0.5 wt%). After shaking for 1 h, the non-encapsulated dye molecules were removed by washing the particles three times with Milli-Q water and subsequently resuspending them in Milli-Q water in an ultrasonic bath. Then, the dye-encapsulated particles were washed with an ethanol−water mixture 50/50 (v/v) to remove dye molecules adsorbed on the particle surface and larger particle aggregates.

Bead encapsulation of dye O and dye I was done with a slightly modified procedure. First, to increase the solubility of the molecules in more hydrophobic solvents or environments, the initially present counter ion perchlorate (ClO_4_^−^) had to be exchanged. Therefore, 100 μL of a dye solution (dye concentration of 2 mM) was added to 0.75 mg lithium tetrakis-(pentafluorophenyl)-borate ethyl etherate (Li-BARF) obtained from Merck GmbH. This solution was then added to 600 μL of a bead dispersion containing 3 mg PS beads (equaling a concentration of 0.5 wt%). Since dyes O and I are insoluble in a 50/50 (v/v) mixture of ethanol and water, after the shaking and washing steps with Milli-Q water, the beads in the particle suspension were allowed to sediment. Subsequently, the dye-stained beads were resuspended in Milli-Q water in an ultrasonic bath. Also, the counter anions periodate (IO_4_^−^), hexafluorophosphate (PF_6_^−^), and tetrafluoroborate (BF_4_^−^) have been tested to increase the solubility of the fluorophores in hydrophobic environments.

### Ensemble steady-state fluorescence spectroscopy

The fluorescence spectra of aqueous bead suspensions were measured in 10 × 10 mm standard cuvettes (Hellma GmbH & Co. KG) with a Spectrofluorometer FluoroMax-4P (HORIBA Jobin Yvon GmbH) equipped with a 150-W ozone-free xenon arc lamp. The emission signals were recorded with a red-sensitive photomultiplier R928P operated in the photon counting mode.

### Fluorescence microscopy and spectroscopy on single bead level

Fluorescence microscopy images of fluorophore-loaded 8 μm PS beads were recorded with a confocal laser scanning microscope (CLSM) FluoView™ FV1000 (OLYMPUS GmbH, Germany). Different excitation light sources were used to excite the encapsulated dyes, thereby considering the different absorption spectra of these fluorophores. Dye C in PS was excited at 355 nm using a DPSS laser Cobolt Zouk® (10 mW), and dye D and dye E in PS were excited at 488 nm and 515 nm, respectively, using a multiline argon ion laser (30 mW). The NIR fluorophore-based CBead O and CBead I were excited with the 633-nm line of a red HeNe-Laser (10 mW). The excitation light was reflected by appropriate dichroic mirrors (DM 351/488/543 or DM 488/543/633) and focused onto the sample through an Olympus objective UPLSAPO 60xW/N.A. 1.2 or UPLSAPO 40x/N.A. 0.9). The emitted fluorescence photons were collected by the same objective and detected in the wavelength region between 400 and 800 nm with photomultiplier tubes (PMTs) in different spectral channels defined by grating monochromators and optical filters.

Microscopic emission spectra of solutions of the KIT dyes F001–F005 and the validation fluorophore Y used for the validation of the spectral correction procedure developed were measured in μ-Slides VI 0.4 (ibidi GmbH). Upon excitation at 355 nm using the laser Cobolt Zouk®, the emission spectra were recorded with an objective UPLSAPO 10x (N.A. 0.40) and corrected for the background emission from the pure solvents ethanol (EtOH) or acetonitrile (ACN) in the case of Y. The fluorophore-loaded candidate calibration beads were suspended in water, transferred onto a coverslip, and measured upon excitation with appropriate excitation wavelengths (see above) with an objective UPLSAPO 60xW (N.A. 1.2). All microscopic emission spectra were detected with a photomultiplier tube (PMT) using a beam splitter BS 20/80, a spectral resolution of 5 nm, and a spectral step size of 2 nm. Both the fluorescence microscopic images and the spatially resolved emission spectra were recorded with sedimented beads suspended in a water droplet that was placed on a standard microscopy coverslip #1.5 (0.170 mm).

## Results and discussion

### Calibration of fluorescence measuring systems with dye solutions

**Fig. 1:**
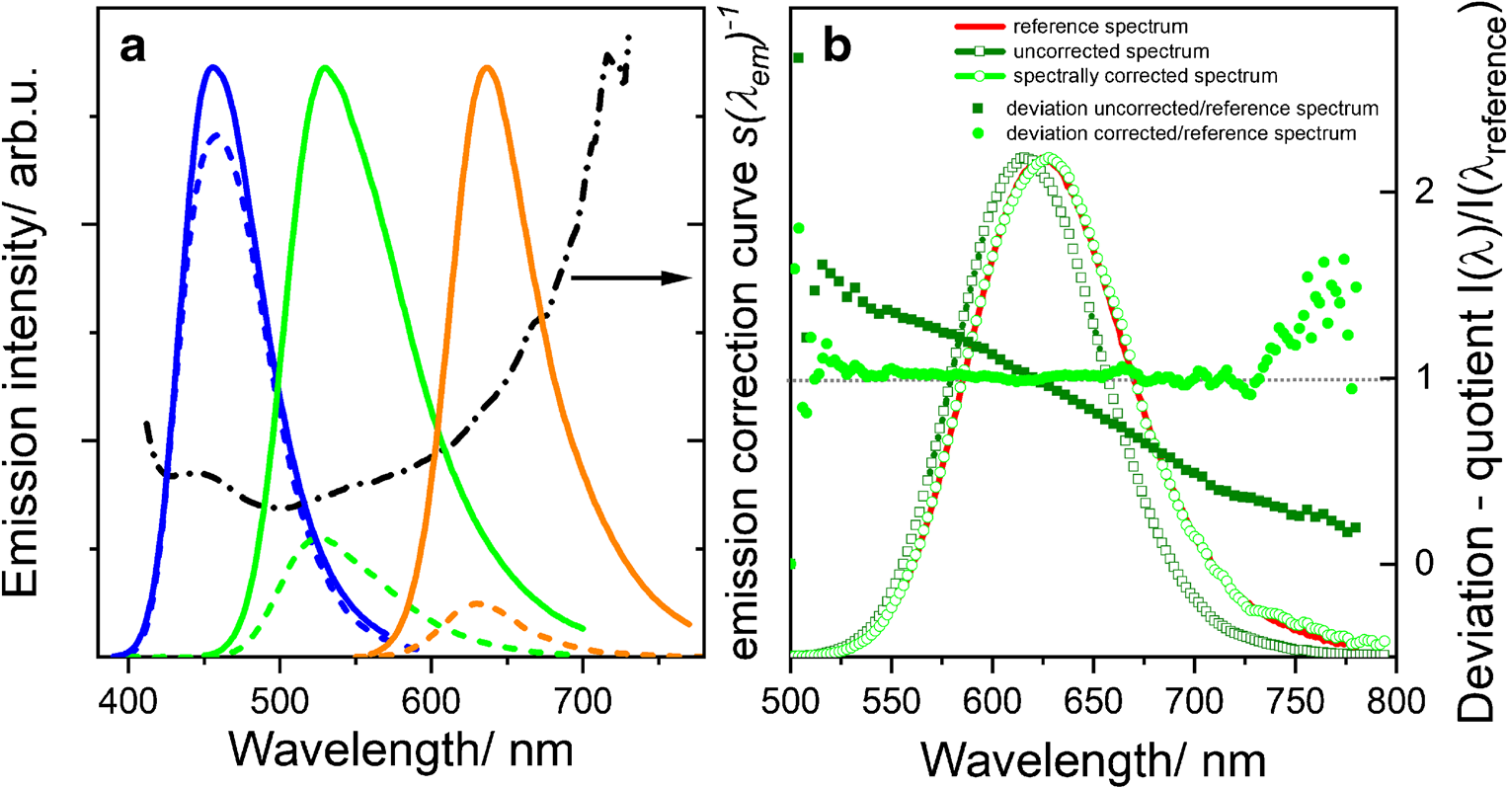
Determination of the spectral characteristics of a fluorescence microscope and its validation. **a** Fluorescence emission spectra of solutions of the 3 spectral fluorescence standards dye C, dye D, and dye E (dashed lines: measured with the CLSM microscope FV1000; solid lines: normalized reference spectra), and the calculated emission correction curve *s*(*λ*_em_)^−1^. **b** Comparison of the emission spectrum of a solution of the validation dye Y [12, 33] as measured with the CLSM FV1000 (olive, open squares) and after fluorophore-based spectral correction (green, open circles) with its spectrally corrected emission spectrum (reference) obtained with a calibrated spectrofluorometer (red line). Solid symbols represent the relative spectral deviations of the uncorrected (solid squares) and spectrally corrected (solid circles) spectra from the latter emission spectrum (red line)

As demonstrated by us, the wavelength-dependent optical characteristics of a spectral scanning microscope, distorting spatially resolved emission spectra [7, 33], can be determined with dye solutions measured in microchannels of μ-Slides VI 0.4. (ibidi GmbH). To assess the spectral characteristics of fluorescence microscopes commonly used for fluorescence measurements from about 400 to 750 nm, only the standard dyes C, D, and E equaling the BAM-certified dyes F003, F004, and F005 of the Kit *Spectral Fluorescence standards* were used in conjunction with the BAM software *LinkCorrWin* [7, 12, 33]. These fluorophores can be excited between 355 and 543 nm with common light sources like lasers and laser diodes used in imaging systems. In addition, the dye solutions were previously assessed regarding a possible dependence of the shape of their emission spectra on typical excitation wavelengths used in fluorescence microscopy [33] and regarding their photostability under prolonged laser excitation. The specifically adapted calibration approach for spectral scanning microscopes, which is illustrated in Fig. 1, can provide the emission correction curve *s*(*λ*_em_)^−1^ from measurements of the dye spectra with the microscope and the dyes’ spectrally corrected emission spectra obtained with a calibrated spectrofluorometer. Multiplication of measured emission spectra with *s*(*λ*_em_)^−1^ then yields device-independent fluorescence spectra, spectrally corrected for microscope-specific distortions. The suitability of this procedure was validated by measuring the uncorrected emission spectrum of a validation dye, here a solution of dye Y in ACN, with this microscope and calculating its corrected emission spectrum with the previously determined emission correction curve *s*(*λ*_em_)^−1^. The resulting corrected emission spectrum was then compared with the spectrally corrected emission spectrum obtained with a calibrated spectrofluorometer. The small relative spectral deviation of the two corrected emission spectra illustrated in the right panel of Fig. 1 underlines the applicability of this dye-based calibration approach to the spectral calibration of microscopes.

### Design of spectral calibration beads—encapsulation of KIT dyes into polymer microparticles

The suitability of the dye-based spectral fluorescence standards from the BAM Kit for the determination of the emission correction curve *s*(*λ*_em_)^−1^ motivated us to exploit these dyes also for the design of candidate bead-based spectral standards. Such calibration beads are much closer to the many rapidly evolving (bio)analytical applications of particulate labels and bead-based platforms, e.g., in suspension microparticle assays [34–39] as well as to eukaryotic and bacteria cells typically measured with fluorescence microscopy techniques than dye solutions. Unlike beads, dye solutions are also not suited for the calibration and IPV of FCM instruments that present another goal of our research focused on the development of calibration and standardization concepts for optical-spectroscopic techniques.

**Fig. 2:**
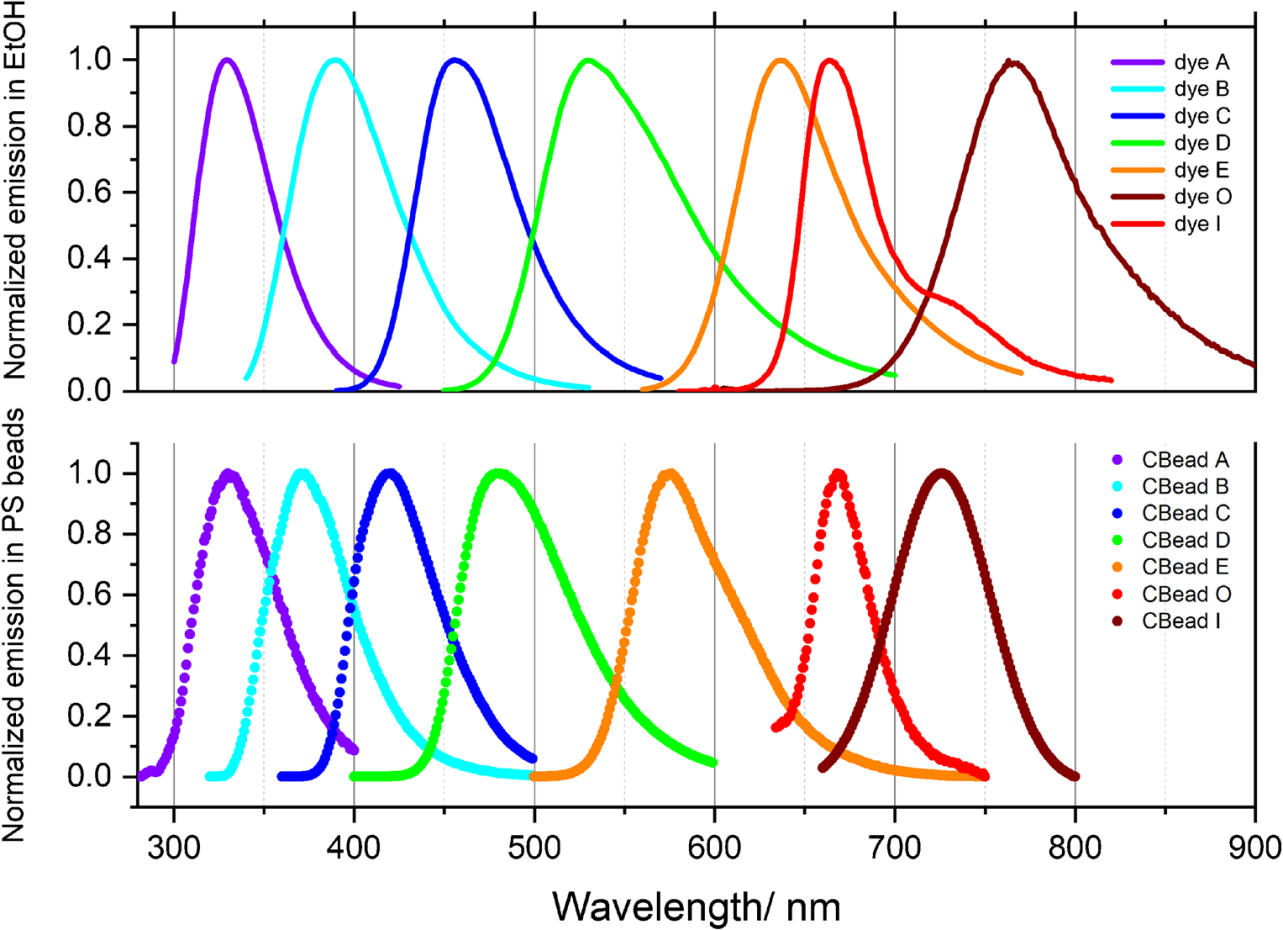
Emission spectra of the BAM Kit dyes A (violet), B (cyan), C (blue), D (green), and E (orange) and the new NIR emissive dyes O (red) and I (brown) in ethanol (top) and encapsulated in 8 μm PS beads (bottom)

**Fig. 3:**
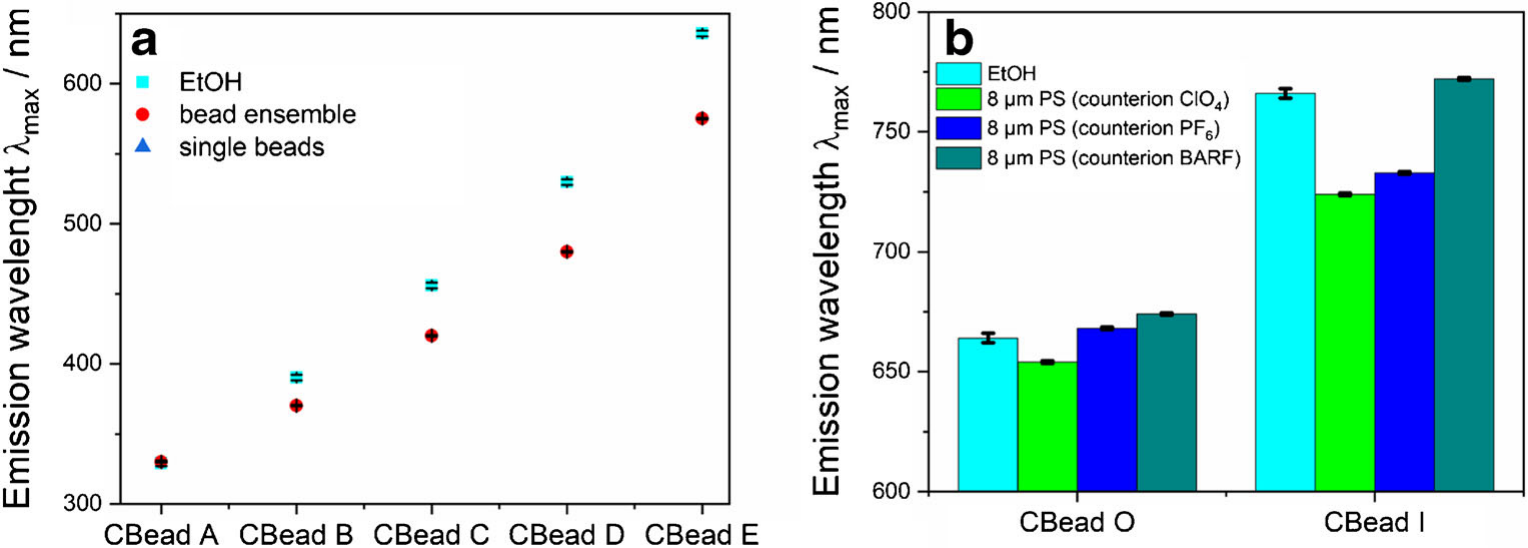
**a** Encapsulation-induced hypsochromic shifts of the emission maxima of the Kit fluorophores in 8-μm PS beads as compared with solutions of the Kit dyes in ethanol underlining the effect of the surrounding matrix on the spectral features of the encapsulated fluorophores; standard deviations derived either from the wavelength accuracy of the respective fluorescence instrument used for the measurements or from the evaluation of single bead spectra (*N* ≥ 5). **b** Influence of the counter anions ClO_4_^−^, PF_6_^−^, and BARF on the spectral position of the emission bands of the CBead O and CBead I. The latter two compounds were obtained from the respective dyes with the perchlorate counter anion by anion exchange

**Fig. 4:**
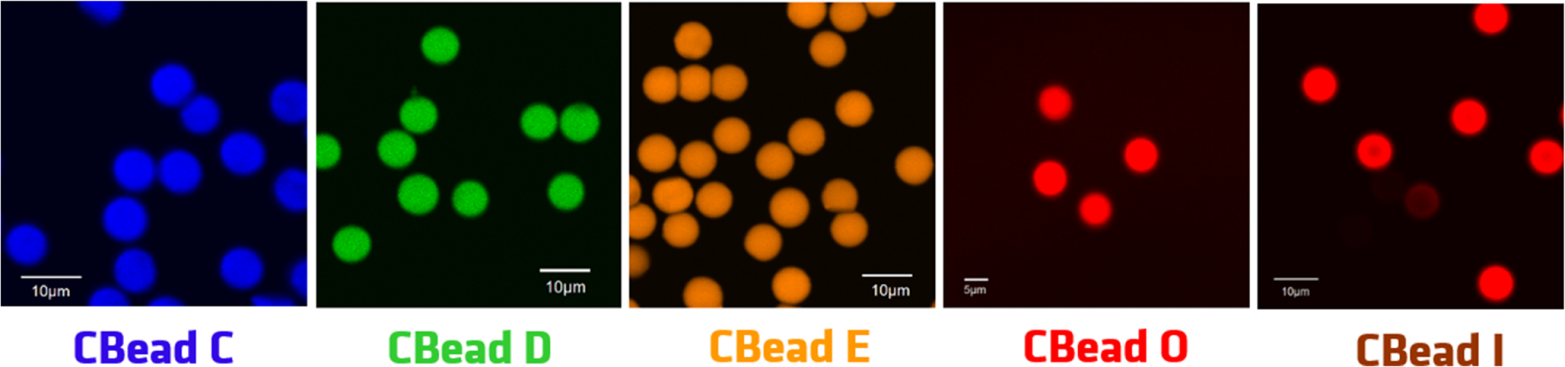
Microscopy images of fluorophore-loaded 8 μm-sized PS beads (UPLSAPO 60x; excitation at 355 nm (CBead C), 488 nm (CBead D), 515 nm (CBead E), and 633 nm (CBead O and CBead I)

The encapsulation approach used requires hydrophobic dyes [29–31] that reveal broad emission spectra also in relatively apolar matrices like PS and cross at sufficiently high fluorescence intensities. The latter is mandatory for the calculation of an overall emission correction curve *s*(*λ*_em_)^−1^ by glueing the individual correction curves calculated for each dye as a quotient of the BAM-certified instrument-independent (corrected) emission spectrum and the measured instrument-specific (uncorrected) emission spectrum as previously derived for the BAM Kit F001–F005 [12]. The main challenge for the development of spectral calibration beads presents the choice of fluorophores that show broad and unstructured emission spectra in an apolar matrix. Broad and unstructured emission spectra are commonly shown by charge transfer (CT)–operated fluorophores like our Kit dyes in polar solvents such as ethanol or ACN. CT dyes are, however, known for the sensitivity of their absorption and emission spectra to polarity. Typically, a decrease in the polarity of dye microenvironment results in a blue shift in absorption and emission and even in the appearance of a slight vibronic fine structure. As a first approach to calibration beads, we incorporated our neutral and hydrophobic Kit dyes A to E [1, 12, 40] (equaling F001 to F005) into premanufactured beads via an established and optimized swelling/deswelling procedure [29–31]. Although the Kit dyes could be straightforwardly incorporated into PS beads at strongly varying dye concentrations (see the corresponding fluorescence microscopy images in Fig. 4 and in Fig. S1 of the Electronic Supplementary Material (ESM)), spectroscopic measurements of the resulting bead ensembles revealed considerable polarity-induced blue shifts in emission compared with the respective dye spectra in ethanol used as solvent for the BAM calibration Kit (see also Table S1 of the ESM). These bead-induced spectral changes are illustrated in Fig. 2 for the emission spectra of the five encapsulated Kit fluorophores. As to be expected, these spectral shifts are particularly pronounced for longer wavelength dyes with a distinct CT character like dye E as highlighted in Fig. 3. These strong PS-induced hypsochromic shifts of the emission bands reduce the wavelength region covered by CBead A to CBead E from about 330 nm to 730 nm in ethanol to about 330 nm to 650 nm in the polymer microparticles.

These matrix-induced blue shifts made the search for new NIR fluorophores necessary to fully cover the wavelength region of 400 to 800 nm relevant for fluorescence microscopy. Screening studies of fluorophores with emission between 600 and 900 nm in polar organic solvents led to the identification of the hydrophobic NIR dye O and dye I. The emission spectra of these new dyes in ethanol are shown in the upper panel of Fig. 2. This figure highlights the desired coverage of the spectral region from about 330 to 900 nm by this set of dye solutions.

### Bead staining with cationic NIR dyes

Subsequently, we tried to encapsulate the hydrophobic and cationic NIR emissive organic dyes O and I with the counterion ClO_4_ into 8 μm-sized PS beads via the same encapsulation procedure as employed previously for the neutral Kit dyes. Homogeneous staining of the PS beads with these dyes was, however, unexpectedly difficult, and failed. To tackle this challenge, we exchanged the initially present counterion ClO_4_^−^ for anions better suited for our bead staining procedure. We tested the anions IO_4_^−^, PF_6_^−^, and BARF (BARF: tetrakis[3,5-bis(trifluoromethyl)phenyl]borate) expected to increase the solubility of these NIR emitters in hydrophobic environments and performed bead loading studies with the resulting dyes varying in counter anion. As criteria for the choice of the optimum counter anions, the emission spectra of the resulting beads and the homogeneity of bead staining were used. The latter was assessed microscopically (see forthcoming section and ESM, Fig. S2). The effect of anion exchange on the spectral position of the emission bands of CBead O and CBead I is displayed in Fig. 3b. This figure also underlines the beneficial red shift in fluorescence introduced by anion exchange which can at least partly counterbalance the bead encapsulation–induced hypsochromic shifts of the emission bands of these NIR fluorophores in the polymer matrix.

### Single bead spectroscopy and microscopy

**Fig. 5:**
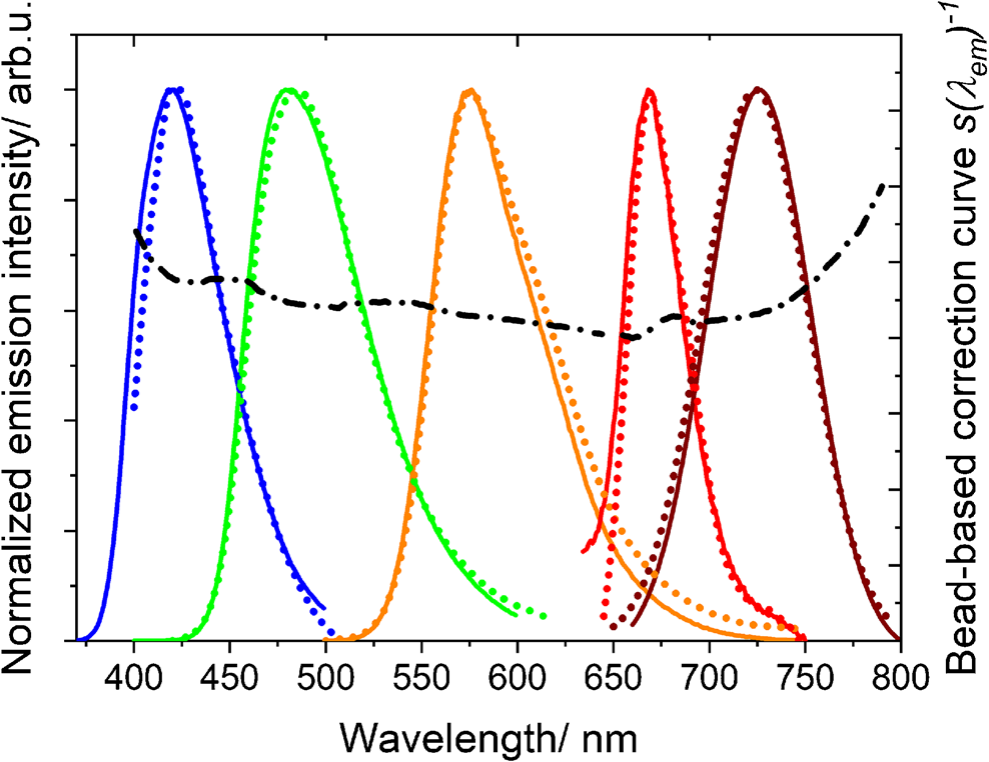
Normalized, spectrally corrected emission spectra of fluorophore-loaded candidate spectral calibration beads CBead C to CBead I (8 μm) obtained from ensemble measurements using a calibrated spectrofluorometer (solid lines) compared with the respective uncorrected emission spectra determined by spectrally resolved microscopy measurements of individual candidate calibration beads (symbols). Evaluation of these data based on the principle of the BAM software *LinkCorrWin* yields the correction curve *s*(*λ*_em_)^−1^ (black dash-dotted line) of the FV1000 microscope

Finally, we studied the emission features of individual PS beads stained with different dyes under optimized conditions, here with focus on the wavelength range of about 400 to 800 nm relevant for fluorescence microscopy, and the homogeneity of dye staining. For this reason, beads loaded with dye A and dye B with emissions below 400 nm were not further used. Figure 4 summarizes the fluorescence microscopy images obtained with 8 μm-sized PS beads stained with dyes C, D, E, O, and I. Bead staining with the neutral Kit dyes C to E leads to a homogeneous distribution of the dye molecules within the beads and high fluorescence intensities with fluorescence quantum yields (QY) obtained for bead ensembles in aqueous dispersion between about 30 and 90% (see ESM, Table S2), which can be easily controlled by dye loading concentration. Staining with the cationic NIR dyes O and I was, however, more challenging as already discussed in the previous section and resulted in QY of 5% and 12%, respectively, and a slightly inhomogeneous fluorophore distribution, even after exchange of the initially present perchlorate anion for the counter anion BARF that reveals the best performance amongst the anions studied (see also ESM, Fig. S2). Nevertheless, the reported staining and anion exchange procedure provides bright candidate calibration beads due to the generally relatively high molar extinction coefficients of NIR dyes, which typically exceed those of organic fluorophores with absorption bands in the visible wavelength region. The normalized emission spectra of the individual candidate calibration beads CBead C to CBead I recorded with our spectral scanning CLSM are shown in Fig. 5 together with the emission spectra obtained for ensembles of suspended beads with a calibrated spectrofluorometer. As follows from this figure, the fluorescence bands of these candidate calibration beads clearly cover the wavelength region from 400 to 800 nm as a set, with the emission spectra of spectrally neighboring fluorophores overlapping as required for the calculation of an overall correction curve with *LinkCorr*. Thus, our criteria for the design of such a kit of spectral calibration beads could be fulfilled.

### Bead-based calibration curves

With these candidate calibration beads in hand, we generated a bead-based spectral correction curve for our CLSM. As reference spectra, we used the spectrally corrected emission spectra of dispersed fluorophore-loaded calibration beads recorded with a calibrated spectrofluorometer. From the quotients of the spectrally corrected reference spectra for each fluorophore-loaded bead population and the instrument-dependent, uncorrected emission spectra measured with the spectral scanning FV1000 microscope, we calculated the microscope-specific emission correction curve *s*(*λ*_em_)^−1^ based on the principle of the BAM software *LinkCorrWin*, distributed with the Kit Spectral fluorescence standards [40]. Using the candidate calibration beads CBead C, CBead D, and CBead E, we could obtain *s*(*λ*_em_)^−1^ in the wavelength region of 400 to 650 nm. By including CBead O and CBead I, this emission correction curve could be extended to almost 800 nm as shown in Fig. 5.

## Conclusion and outlook

We developed a set of candidate spectral calibration beads for the reliable characterization of the spectral characteristics of fluorescence microscopes under routine measurement conditions by incorporating hydrophobic dyes with broad emission bands into micrometer-sized polystyrene (PS) beads via a previously established staining procedure. Challenges to overcome during the development of this set of candidate spectral bead standards included matrix-induced blue shifts of the emission spectra of polarity-sensitive charge transfer dyes, the identification of new NIR emitters, and the choice of suitable counter anions for an improved bead staining. The emission spectra of the final set of dye-stained beads cover the wavelength range from 400 to 800 nm. This was accomplished by combining the neutral dyes from the BAM-certified Kit Spectral Fluorescence Standards with two newly identified near-infrared (NIR) emissive cationic dyes with the counterion tetrakis[3,5-bis(trifluoromethyl)phenyl]borate (BARF). The bead set developed and shown here presents the first step towards a new platform of spectral calibration beads for the determination of the spectral characteristics of fluorescence measuring devices like fluorescence microscopes, FCM setups, and microtiter plate readers, thereby meeting the increasing demand for reliable and comparable fluorescence data especially in strongly regulated areas like, e.g., medical diagnostics [41].

Our results provide an important step towards an improved comparability of fluorescence data, across different instruments, laboratories, and even methods with special emphasis on spectroscopic methods studying micrometer-sized fluorescent beads or objects or relying on their use such as bead-based assays. Moreover, as shown for example by H. Zhou et al. [42], NIR-bead standards are relevant not only for fluorescence microscopy but also for flow cytometry where applications of NIR flow cytometry have been emerging in the last years. Further experiments are currently focused on the screening of other dyes to identify fluorophores that even better meet the stringent requirements imposed by us on sets of spectral standards with overlapping emission spectra. This is relevant for the calculation of overall spectral correction curves for the whole emission region covered by the fluorescence spectra of the dyes as previously derived for the dyes of the BAM Calibration Kit Spectral Fluorescence Standards [12]. Such spectral calibration beads, which can be produced in different sizes and intensities, can be also incorporated as internal standards, e.g., into microfluidic devices, sensor arrays, or strips used for lateral flow assays or can be printed to generate nano- or micro-structured calibration tools.

## Electronic supplementary material

ESM 1 (PDF 301 kb).
