# Supplementary material for: Fluorescence calibration standards made from broadband emitters encapsulated in polymer beads for fluorescence microscopy and flow cytometry
Source: Anal Bioanal Chem. 2020 May 14;412(24):6499–507. doi: 10.1007/s00216-020-02664-y (PMC7442758; doi:10.1007/s00216-020-02664-y)
Supplement: Supplementary file 1 — (PDF 301 kb). [file 216_2020_2664_MOESM1_ESM.pdf]

Analytical and Bioanalytical Chemistry

Electronic Supplementary Material

Fluorescence calibration standards made from broadband emitters encapsulated in polymer beads for fluorescence microscopy and flow cytometry

Katrin Hoffmann, Nithiya Nirmalananthan-Budau, Ute Resch-Genger

Content

1. Control images of non-modified beads (**Figure S1**)
2. Fluorescence data of candidate calibration beads in ensemble and at single particle level (**Table S1**)
3. Information on fluorescence quantum yields and on bead staining homogeneity (**Table S2**)
4. Microscopic investigation of candidate calibration beads (**Figure S2**)

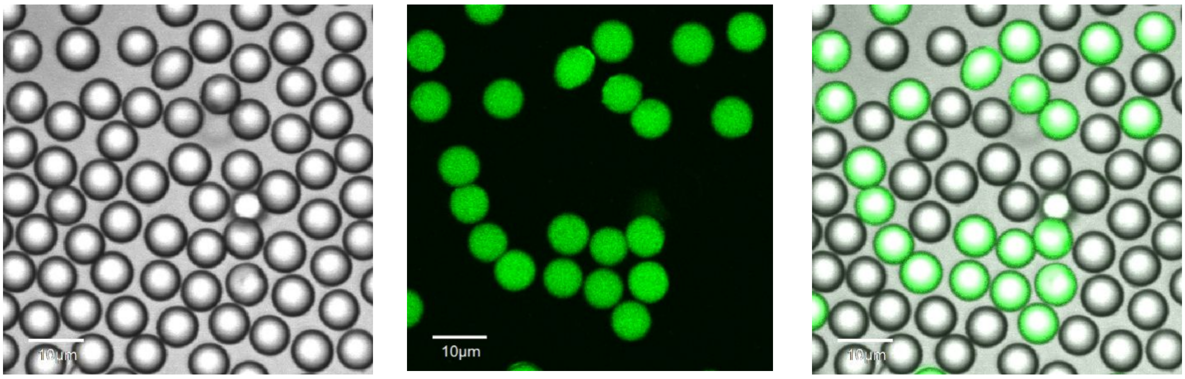

Fig. S1 Control images of an exemplary mixture of non-modified polystyrene (PS) beads (8 µm) and PS beads with encapsulated Dye D: transmission image (left), fluorescence image (middle) and their overlay (right). No emission signal can be detected from the non-modified PS beads

Table S1 Fluorescence data (emission wavelengths) of dyes measured in different environments, in ethanolic solution, and in 8 µm polystyrene (PS) beads in aqueous suspensions in the ensemble and at single particle level

		emission wavelength λ_{max}/nm	
fluorophore	EtOH	8 µm PS (ensemble)	8 µm PS (single bead level)
DyeA	329±2	330±0.5	n.d.
DyeB	390±2	370±0.5	n.d.
DyeC	456±2	420±0.5	422±0.8
DyeD	530±2	480±0.5	485±2
DyeE	636±2	575±0.5	576±0.8
DyeO ClO4	664±2	668±0.5	669±3.6
DyeO ClO4 US	-	654±0.5	-
DyeO PF6	-	668±0.5	-
DyeO BARF	-	673±0.5	-
DyeO BARF US	-	672±0.5	678±0.8
DyeI ClO4	766±2	726±0.5	719±0.5
DyeI ClO4	-	724±0.5	-
DyeI PF6	-	733±0.5	-
DyeI BARF	-	772±0.5	742±0.8

*) Standard deviations derived from the wavelength accuracy of the fluorescence instrument
**) Standard deviations derived from evaluation of single bead spectra (N ≥ 5)
***) US: ultrasound

Table S2 Fluorescence quantum yields (QY) of dispersions of dye-encoded 8 µm polystyrene (PS) beads absolutely measured with a C11347 (Hamamatsu Photonics K.K.) integrating sphere setup (error represents the standard deviation (SD) of twelve independent measurements). The integral and average intensities of different bead-related regions of interest (ROI) of the dyes-stained bead batches were obtained to estimate bead staining homogeneity

fluorophore	Quantum yield/ %		Staining homogeneity (integral bead intensity)	
	λ_{exc}/nm	8 µm PS (ensemble)	ROI integration	ROI average
DyeC	355	87±3	5.40E+06±3.28E+05	2.64E+03±1.60E+02
DyeD	488	31±2	2.99E+06±1.61E+05	1.98E+03±1.02E+02
DyeE	515	66±2	5.29E+06±3.05E+05	2.87E+03±1.66E+02
DyeO*)	633	5±1	5.11E+06±1.08E+06	1.94E+03±4.08E+02
DyeI*)	633	12±1	4.17E+06±1.74E+06	2.33E+03±9.72E+02

*) counterion ClO_4

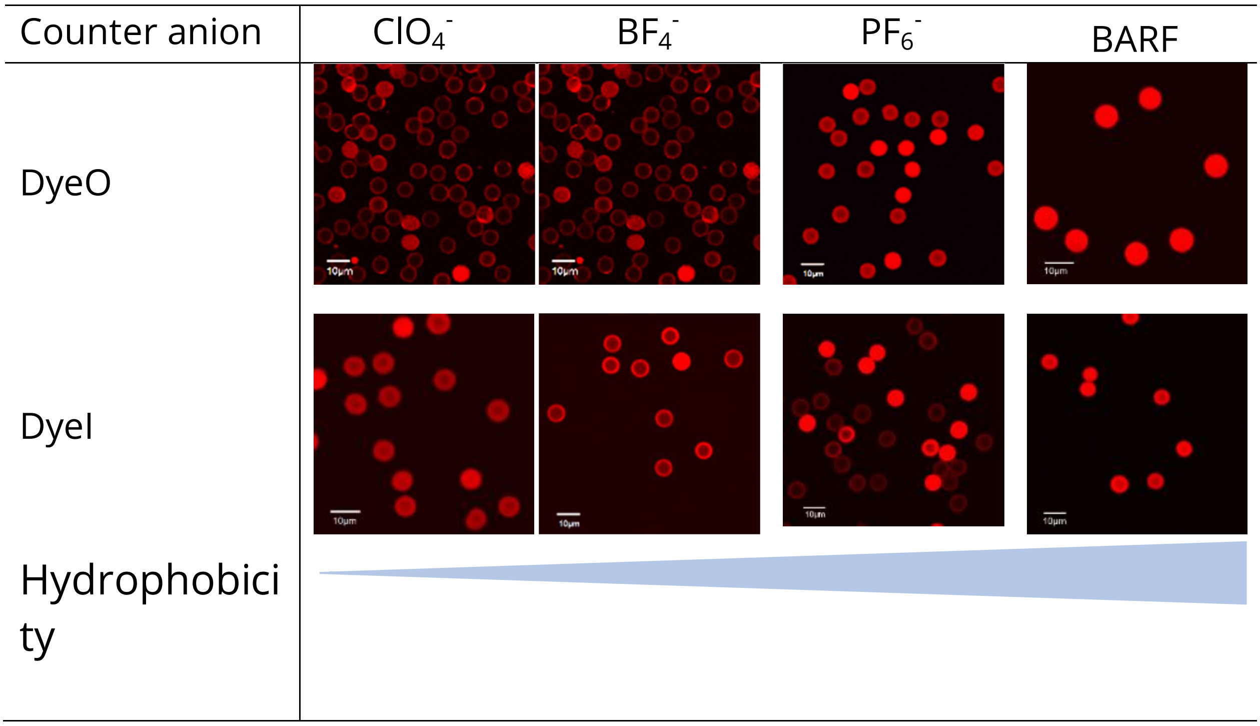

Fig. S2 Visual, microscopic investigation of candidate calibration beads during the course of counter anion exchange studies (confocal laser scanning Microscope OLYMPUS FV1000; UPLSAPO 40x/0.9; excitation wavelength 633 nm)
